# Longitudinal monitoring of autoantibody dynamics in patients with early-stage non-small-cell lung cancer undergoing surgery

**DOI:** 10.1515/biol-2025-1133

**Published:** 2025-10-18

**Authors:** Jinming Miao, Zhitong Wang, Lin Li, Shuai Pei, Jichao Chu, Bingshan Guo, Xingchen Li, Yudan Zheng, Yongzhi Wang

**Affiliations:** Department of Thoracic Surgery, Dandong First Hospital, 76 Baoshan Street, Dandong, Liaoning, 118000, China; Department of Thoracic Surgery, The First Hospital of China Medical University, 76 Baoshan Street, Shenyang, Liaoning, 110001, China

**Keywords:** lung cancer, autoantibodies, recurrence, survival, biomarkers

## Abstract

Lung cancer is one of the most common and deadly malignancies worldwide, underscoring the need for reliable biomarkers that can inform prognosis and guide postoperative surveillance. This prospective study examined longitudinal changes in 10 tumor-associated autoantibodies in 71 patients with early-stage non-small-cell lung cancer (NSCLC) who underwent surgical resection. Blood samples were collected preoperatively and at 3-, 6-, and 12-month post-surgery. Enzyme-linked immunosorbent assays were used to measure serum autoantibodies against p53, MUC1, NY-ESO-1, APE1, PGP9.5, SOX2, GBU4-5, GAGE7, CAGE, and MAGE1. Logistic regression models assessed associations with 1-year recurrence, while Cox proportional hazards models evaluated overall survival. Substantial reductions in p53, GBU4-5, and CAGE autoantibodies correlated with lower recurrence risk and improved 1-year survival, even after false discovery rate adjustment (*p* < 0.05). NY-ESO-1 showed borderline significance for recurrence, and SOX2 was borderline for survival but did not remain significant after correction. These findings suggest that monitoring dynamic declines in certain autoantibodies (most notably CAGE) may offer clinically meaningful prognostic information following surgical resection. While further validation in larger, independent cohorts is required, our results highlight the potential of serial autoantibody profiling as a noninvasive tool for personalized postoperative management in early-stage NSCLC patients.

## 1 Introduction

Lung cancer remains the foremost cause of cancer‑related death worldwide, placing a considerable burden on public health [1]. Recent advances in computed tomography screening have increased the detection of early‑stage disease, thereby shifting the overall spectrum of lung‑cancer diagnoses toward earlier stages [2]. Surgical resection – most commonly lobectomy – remains the standard of care for early‑stage non‑small-cell lung cancer (NSCLC) [3]. Emerging evidence suggests that anatomic segmentectomy can deliver oncologic outcomes comparable to lobectomy in appropriately selected patients [4].

Postoperative prognosis now hinges increasingly on advanced surveillance modalities and predictive modeling. Circulating tumor DNA has emerged as a sensitive biomarker for minimal residual disease, enabling early identification of relapse risk [5]. Low-dose computed tomography surveillance protocols further improve recurrence-free and overall survival (OS) compared with conventional CT [6]. Concurrently, machine-learning algorithms are being developed to generate individualized predictions of postoperative life expectancy and complication risk [7,8]. Together, these innovations promise to refine post-surgical management and enhance long-term outcomes for patients undergoing lung-cancer resection.

Tumor‑associated antigens (TAAs) displayed on cancer‑cell surfaces can trigger host immune responses, generating circulating autoantibodies that are now recognized as promising biomarkers for cancer diagnosis. Although no FDA-approved autoantibody test for lung cancer, a commercial test with a seven-autoantibody assay (EarlyCDT-Lung) already exists and has been evaluated in technical-validation studies, nodule-management cohorts, and the large early detection of cancer of the Lung Scotland (ECLS) randomized trial [9,10,11]. A five‑autoantibody panel has been shown to outperform conventional markers such as CA 19‑9 in pancreatic ductal adenocarcinoma [12]. Likewise, multiplex panels targeting several TAAs markedly enhance early lung‑cancer detection, with certain combinations achieving up to 65% sensitivity while maintaining 100% specificity [13]. A multicenter Chinese study developed a nine-marker “CN9” autoantibody panel that improved nodule-malignancy discrimination when added to Mayo or Brock risk models [14]. More recently, Guo et al. reported that a seven-autoantibody signature, combined with machine-learning, achieved an AUC ≈ 0.93 for ground-glass nodular adenocarcinoma (≤3 cm) [15].

Autoantibodies have also emerged as promising biomarkers for disease progression, recurrence, and survival across various cancer types. In gastrointestinal malignancies, autoantibodies against p53 and NY-ESO-1 track with tumor progression – particularly in esophageal squamous-cell carcinoma and gastric cancer – underscoring their prognostic potential [16]. In gastric cancer, serum NY-ESO-1 and p53 autoantibodies are associated with postoperative recurrence, with NY-ESO-1 seropositivity at 3 and 12 months after surgery independently predicting shorter recurrence-free survival [17]. Elevated titers of multiple autoantibodies likewise correlate with prognosis in lung cancer [18,19]. Although a recent study documented postoperative shifts in the autoantibody repertoire of lung-cancer patients [20], the influence of these changes on long-term outcomes after resection remains largely unexplored.

This study will characterize the peri‑operative kinetics of a ten‑autoantibody panel – p53, MUC1, NY‑ESO‑1, APE1, PGP9.5, SOX2, GBU4‑5, GAGE7, CAGE, and MAGE1 – in patients undergoing lung‑cancer resection. By mapping postoperative trajectories and correlating them with recurrence, metastasis, and survival, we aim to elucidate each autoantibody’s prognostic value. If validated, such profiling could be incorporated into routine follow‑up as a non‑invasive, personalized surveillance tool to inform adjuvant‑therapy decisions and improve long‑term outcomes.

## 2 Materials and methods

**Figure 1:**
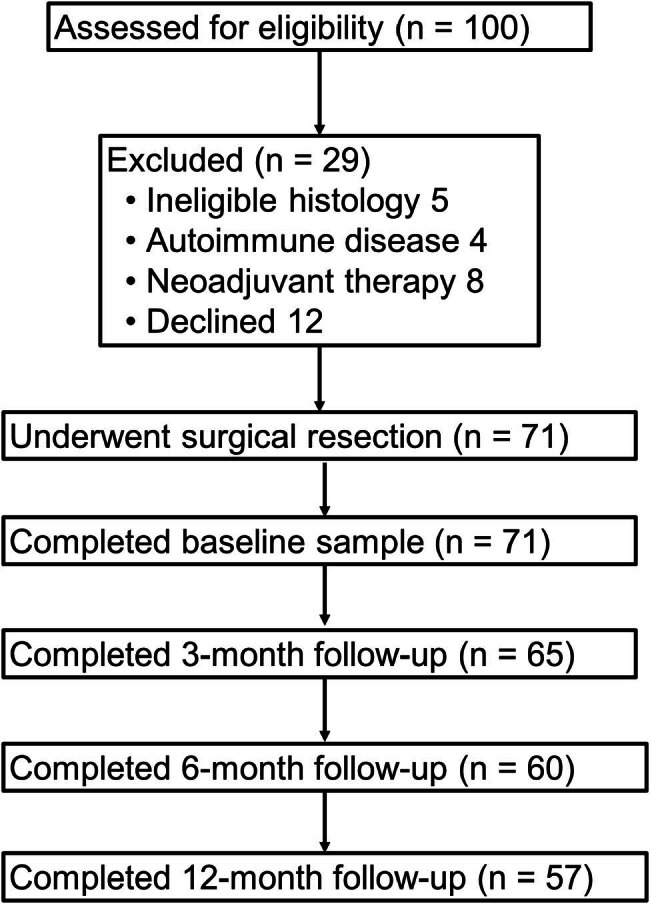
Flowchart of participant selection.

This study was a prospective observational cohort study conducted at Dandong No. 1 Hospital from August 2019 to December 2023. Patients with confirmed primary lung cancer scheduled for surgical resection were enrolled based on predefined inclusion and exclusion criteria. All patients were treated with an identical cisplatin + pemetrexed regimen; no radiotherapy or immunotherapy was administered during the first postoperative year. Eligible participants were adults (≥18 years) with histologically confirmed primary NSCLC who were considered suitable for curative surgical resection. Patients were excluded if they had a prior malignancy, had received neoadjuvant therapy, or had autoimmune disorders or other conditions known to alter autoantibody levels (Figure 1).

### 2.1 Sample size considerations

No formal statistical power calculation was performed for this exploratory study. We aimed to enroll consecutive, eligible patients until we achieved a sufficiently sized cohort (*n* ≥ 70) that would permit preliminary subgroup and regression analyses. This number was guided by practical feasibility and prior literature on autoantibody biomarker studies in early-stage lung cancer, which often involve similar sample sizes.


**Informed consent:** Informed consent has been obtained from all individuals included in this study.
**Ethical approval:** The research related to human use has been complied with all the relevant national regulations, institutional policies, and in accordance with the tenets of the Helsinki Declaration, and has been approved by the Institutional Review Board of Dandong First Hospital (DDSDYYY-2022-08-20-01).

### 2.2 Performance status

Prior to surgery, each patient’s functional capacity was assessed using the ECOG Performance Status scale. Patients with ECOG PS 0–2 were considered suitable candidates for curative-intent surgical resection, in line with institutional guidelines. Consequently, all 71 enrolled patients had ECOG PS ≤2 at baseline.

### 2.3 Sample collection and laboratory measurements

#### 2.3.1 Time points

Patients underwent scheduled blood draws at four time points: baseline (pre-surgery), 3, 6, and 12 months post-surgery. These time points were selected to capture the perioperative immune response and its evolution over time.

#### 2.3.2 Blood sample processing

Blood samples were collected into vacutainer tubes and allowed to clot at room temperature for 30 min. Serum was separated by centrifugation at 3,000 rpm for 10 min and immediately aliquoted into cryogenic vials. All serum samples were stored at −80°C until analysis.

#### 2.3.3 Autoantibody-panel selection rationale

The ten TAAs assayed in this study – p53, MUC1, NY-ESO-1, APE1, PGP9.5, SOX2, GBU4-5, GAGE7, CAGE, and MAGE1 – were chosen *a priori* using three complementary criteria: (i) reproducible over-expression or mutation in NSCLC tissue together with seropositivity ≥20% in at least one independent cohort [13,21,22]; (ii) mechanistic representation of key oncogenic processes – genomic instability/p53, aberrant mucin glycosylation/MUC1, cancer-testis antigenicity/NY-ESO-1 and MAGE1, DNA-repair dysregulation/APE1, neuronal-protein re-expression/PGP9.5 and GAGE7, developmental transcription factors/SOX2, and stress-induced chaperone-like proteins/GBU4-5 and CAGE [13]; and (iii) prior evidence that circulating autoantibodies to the antigen aid early detection or predict postoperative recurrence or survival in at least two external studies [13,19,23]. Limiting the panel to these ten well-validated TAAs balanced biological diversity with assay practicality and avoided redundancy while excluding markers such as HER2, survivin, and ENO1, whose published seroprevalence in early-stage NSCLC is <10% or whose longitudinal prognostic performance is inconsistent [22,24].

#### 2.3.4 Autoantibody assays

Autoantibody levels for P53, MUC1, NY-ESO-1, APE1, PGP9.5, SOX2, GBU4-5, GAGE7, CAGE, and MAGE1 were measured in duplicate using indirect enzyme-linked immunosorbent assays (ELISA). Purified human recombinant proteins (TP53, MUC1, NY-ESO-1, APE1, PGP9.5, SOX2, GBU4-5, GAGE7, CAGE, and MAGE1) were dissolved to 0.25 µg/µL. Each protein, diluted in coating buffer (50 mM sodium carbonate/bicarbonate, pH 9.6), was added at 100 µL per well to 96-well plates and incubated overnight at 4°C. Human IgG (#I4506, Sigma-Aldrich) was diluted (300–0 ng/mL) to create standard curves. After blocking with 2% BSA overnight at 4°C, plates were washed three times with PBST (PBS + 0.05% Tween 20). Serum samples (diluted 1:100) were added (100 µL/well) and incubated at 37°C for 1 h. Following five washes, 100 µL of Rec-Protein A-HRP (Cat#101123, Invitrogen, 1:10,000 in 1% BSA) was added and incubated at 37°C for 1 h. After another five washes, tumor mutational burden substrate was added, and the reaction was stopped with 2 M H_2_SO_4_. Optical density was read at 450 nm, and autoantibody concentrations were calculated from the IgG standard curve. Positive and negative controls for each tumor-associated autoantibody were included on each plate for quality control.

We estimated assay sensitivity (limit of detection) by testing decreasing concentrations of human IgG reference standard. For each autoantibody, both high-positive and low-positive controls were analyzed in duplicate within the same plate and across different plates. The average intra-assay coefficient of variation (CV) ranged from 5 to 8%, and the inter-assay CV ranged from 7 to 10% across all markers, meeting established quality control standards for clinical research ELISAs. Positive, negative, and blank (no-antigen) controls were included on each 96-well plate.

## 3 Clinical assessments and follow-up

### 3.1 Outcome measures

- **Recurrence**: It is defined as radiological or histopathological evidence of new tumor growth at the surgical bed or within the ipsilateral lung. Surveillance CT or PET‑CT scans were obtained at protocol‑specified intervals, and suspicious lesions were confirmed histologically whenever feasible.
- **Metastasis**: Diagnosed when cross‑sectional imaging (CT, MRI, or PET‑CT) and clinical assessment demonstrate tumor deposits at distant sites. Follow‑up evaluations were performed every 3 months during the first postoperative year and subsequently at routine clinic visits.
- **Survival**: OS was the interval from the date of surgery to death from any cause; disease‑specific survival was the interval from surgery to death attributable to lung cancer. Vital status was ascertained through electronic medical records and telephone follow‑up.

### 3.2 Data collection

Demographic, clinical, and histopathologic variables – including age, sex, smoking history, tumor stage, and histologic subtype – were abstracted from the electronic medical record. Follow‑up information (imaging reports, clinical notes, and laboratory results) was prospectively documented at scheduled postoperative visits. All data were entered into a secure, de‑identified database to ensure patient confidentiality.

### 3.3 Statistical analysis

Descriptive statistics summarized patient demographics, baseline clinical features, and autoantibody concentrations. Continuous variables are reported as means ± standard deviations, whereas categorical variables are expressed as counts and percentages. Pearson correlation coefficients were calculated to explore associations between autoantibody levels and clinical outcomes, including recurrence and metastasis. Univariable and multivariable logistic regression models were constructed to identify autoantibodies independently associated with adverse events – recurrence, metastasis, and OS – while adjusting for age, sex, and tumor stage. Statistical significance was set at *P* < 0.05.

## 4 Results

### 4.1 Demographic and baseline autoantibody profiles in lung cancer patients

Table 1 summarizes the demographic features and baseline autoantibody profiles of the 71 patients who underwent lung‑cancer resection. The cohort’s mean age was 61.7 ± 9.9 years, and women outnumbered men (60.6 vs 39.4%). Mean body‑mass index was 22.4 kg m^−2^. Baseline serum concentrations for each of the ten autoantibodies are also listed.

**Table 1:** Demographic characteristics and baseline autoantibody levels

Characteristic	Value
**Age (years)**, mean ± SD	61.7 ± 9.9
**Sex**	
Male, *n* (%)	28 (39.4%)
Female, *n* (%)	43 (60.6%)
**Body Mass Index**, mean ± SD	22.4 ± 3.2
**Smoking Status**	
Current/Ex-smoker, *n* (%)	14 (19.7%)
Never, *n* (%)	57 (80.3%)
**Tumor Stage**, *n* (%)	
Stage I	32 (45.1%)
Stage II	39 (54.9%)
**Histology**, *n* (%)	
Adenocarcinoma	69 (97.2%)
Squamous cell carcinoma	1 (1.4%)
Adenosquamous carcinoma	1 (1.4%)
**Comorbidities**	
Hypertension, *n* (%)	18 (25.3%)
Diabetes Mellitus, *n* (%)	12 (16.9%)
**Autoantibody Baseline Levels (U/mL)**, mean ± SD	
P53	18.5 ± 5.2
MUC1	22.1 ± 4.8
NY-ESO-1	12.6 ± 3.5
APE1	14.9 ± 4.2
PGP9.5	16.3 ± 3.9
SOX2	13.4 ± 4.1
GBU4-5	18.9 ± 3.3
GAGE7	11.5 ± 3.2
CAGE	14.3 ± 3.8
MAGE1	17.6 ± 4.9

Most participants were never‑smokers (57/71, 80.3%), while 14 (19.7%) were current or former smokers. At diagnosis, 32 patients (45.1%) had stage I disease and 39 (54.9%) had stage II. Adenocarcinoma was overwhelmingly predominant (69/71, 97.2%); squamous‑cell carcinoma and adenosquamous carcinoma each accounted for one case (1.4% per subtype).

Baseline serum autoantibody concentrations (mean ± SD, U mL^−1^) were: P53 (18.5 ± 5.2), MUC1 (22.1 ± 4.8), NY-ESO-1 (12.6 ± 3.5), APE1 (14.9 ± 4.2), PGP9.5 (16.3 ± 3.9), SOX2 (13.4 ± 4.1), GBU4-5 (18.9 ± 3.3), GAGE7 (11.5 ± 3.2), CAGE (14.3 ± 3.8), and MAGE1 (17.6 ± 4.9) (Table 1).

### 4.2 Dynamics of autoantibody responses post-lung cancer surgery

Table 2 depicts serial autoantibody kinetics from baseline through 3, 6, and 12 months after resection. p53 titers rose modestly at 3 months, then declined sharply at 6 months and continued to fall at 12 months. A similar “early‑rise, late‑fall” pattern was evident for SOX2, MUC1, and MAGE1. NY‑ESO‑1 reached a more subdued peak at 3 months before tapering gradually over subsequent visits. Collectively, these trajectories underscore a dynamic postoperative immune landscape, suggesting that shifts in tumor micro‑environment or host immune surveillance evolve over the first postoperative year.

**Table 2:** Autoantibody level changes over time

Autoantibody	Baseline	3 months	*p*-value vs baseline	6 months	*p*-value vs baseline	12 months	*p*-value vs baseline
p53	18.5 ± 5.2	20.1 ± 5.4	0.050	13.7 ± 4.2	0.001	5.5 ± 0.8	<0.001
GBU4-5	18.9 ± 3.3	17.2 ± 3.5	0.044	12.4 ± 3.3	0.010	7.1 ± 1.1	<0.001
CAGE	14.3 ± 3.8	13.7 ± 3.4	0.012	9.1 ± 3.2	0.010	6.4 ± 2.9	<0.001
NY-ESO-1	12.6 ± 3.5	11.7 ± 3.8	0.045	7.8 ± 3.1	0.050	4.5 ± 0.9	<0.001
SOX2	13.4 ± 4.1	14.3 ± 3.9	0.314	8.7 ± 3.6	0.005	5.0 ± 1.8	<0.001
MUC1	22.1 ± 4.8	24.1 ± 4.5	0.043	14.9 ± 3.9	0.080	6.3 ± 1.7	0.010
APE1	14.9 ± 4.2	14.2 ± 3.7	0.651	11.2 ± 3.5	0.120	8.8 ± 1.0	0.020
PGP9.5	16.3 ± 3.9	16.1 ± 4.1	0.247	10.8 ± 3.6	0.050	5.2 ± 2.4	0.010
GAGE7	11.5 ± 3.2	10.8 ± 2.9	0.062	8.9 ± 2.7	0.080	4.8 ± 1.4	0.020
MAGE1	17.6 ± 4.9	18.4 ± 4.9	0.085	9.6 ± 4.0	0.050	5.1 ± 2.5	0.010

### 4.3 Logistic-regression analysis of factors influencing recurrence in lung cancer patients

**Figure 2:**
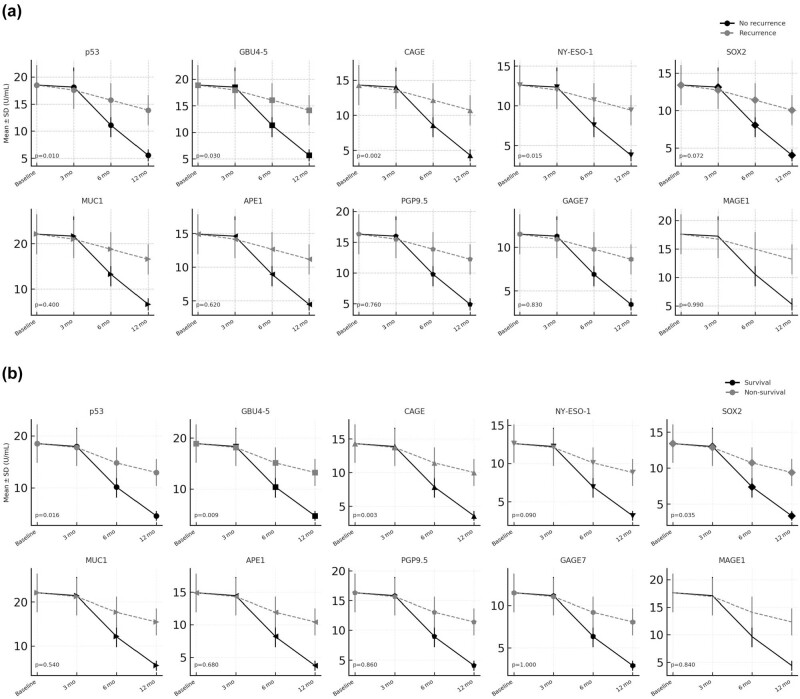
Longitudinal Autoantibodies Dynamics. (a) Longitudinal distribution of autoantibody titers after resection. (b) Individual postoperative trajectories of autoantibodies across time‑points.

Autoantibodies dynamic in recurrence and non-recurrence patients were presented in Figure 2a. Univariable logistic‑regression analysis showed that postoperative reductions in several autoantibody titers were significantly associated with the odds of recurrence; these associations remained robust after adjustment for age, sex, tumor stage, and smoking status in the multivariable model (Table 3).

**Table 3:** Univariable and multivariable analyses for 1-year recurrence

Variable	Univariable			Multivariable		
	OR (95% CI)	*p*	FDR *p*	OR (95% CI)	*p*	FDR *p*
p53 (≥50% reduction)	0.50 (0.30–0.85)	0.010	0.065	0.55 (0.32–0.95)	0.032	0.058
GBU4-5 (≥45% reduction)	0.56 (0.33–0.95)	0.030	0.078	0.60 (0.35–1.02)	0.058	0.058
CAGE (≥50% reduction)	0.42 (0.25–0.72)	0.002	0.026	0.48 (0.27–0.82)	0.007	0.035
NY-ESO-1 (≥50% reduction)	0.52 (0.31–0.88)	0.015	0.065	0.55 (0.31–0.96)	0.039	0.058
SOX2 (≥45% reduction)	0.60 (0.34–1.05)	0.072	0.134	—	—	—
MUC1 (≥40% reduction)	0.80 (0.47–1.35)	0.400	0.650	—	—	—
APE1 (≥40% reduction)	0.88 (0.52–1.47)	0.620	0.806	—	—	—
PGP9.5 (≥50% reduction)	0.92 (0.54–1.56)	0.760	0.897	—	—	—
GAGE7 (≥50% reduction)	0.95 (0.57–1.58)	0.830	0.899	—	—	—
MAGE1 (≥50% reduction)	1.00 (0.60–1.66)	0.990	0.990	—	—	—
Age (per 1-year increase)	1.03 (0.99–1.07)	0.070	0.152	—	—	—
Stage II vs Stage I	1.30 (1.02–2.30)	0.021	0.068	1.25 (0.99–2.09)	0.055	0.058
Sex (female vs male)	0.85 (0.48–1.53)	0.600	0.867	—	—	—
Smoking (current/ex vs never)	1.12 (0.62–2.05)	0.690	0.812			
Hypertension	1.05 (0.56–1.98)	0.870	0.899			
Diabetes	1.21 (0.60–2.46)	0.590	0.756			
Charlson ≥ 2	1.18 (0.63–2.23)	0.610	0.767			

In the univariate analysis, significant associations were found for reductions in several autoantibodies. p53 (≥50% reduction), GBU4-5 (≥45% reduction), CAGE (≥50% reduction), and NY-ESO-1 (≥50% reduction) each showed a significant association with lower recurrence risk (raw *p* < 0.05), after applying the Benjamini–Hochberg correction for multiple tests, CAGE remained significant (false discovery rate [FDR] *p* = 0.026), while p53, GBU4-5, and NY-ESO-1 were borderline (Table 3). The multivariate analysis, which adjusts for other variables, only CAGE retained significance as the most robust predictor of lower recurrence, retaining statistical significance after FDR adjustment (FDR *p* = 0.035). Notably, other autoantibodies did not show significant associations in either univariate or multivariate models (Table 3).

### 4.4 Prognostic significance of autoantibody reductions in lung cancer survival

Autoantibodies dynamic in survival and non-survival patients are presented in Figure 2b. Cox regression analysis was conducted to evaluate the impact of autoantibody reductions on OS in lung cancer patients (Table 4). In the univariate analysis, p53 (≥50% reduction), GBU4-5 (≥45% reduction), and CAGE (≥50% reduction) demonstrated significant associations with improved 1-year OS (raw *p* < 0.05), With FDR correction, only CAGE remained below the 0.05 threshold (FDR *p* = 0.039) (Table 4). In the multivariate model, CAGE (FDR *p* = 0.020), GBU4-5 (FDR *p* = 0.034), and p53 (FDR *p* = 0.045) all achieved significance, indicating that reductions in these autoantibodies are each associated with improved 1-year survival (Table 4). By contrast, SOX2 remained only borderline significant (raw *p* = 0.070, FDR *p* = 0.070) when included and was eventually excluded from the final model. These results highlight CAGE as a particularly strong marker of survival benefit, with additional support for GBU4-5 and p53 once multiple testing is taken into account.

**Table 4:** Univariable and multivariable analyses for 1-year OS

Variable	Univariable			Multivariable		
	HR (95% CI)	*p*	FDR *p*	HR (95% CI)	*p*	FDR *p*
p53 (≥50% reduction)	0.58 (0.37–0.90)	0.016	0.069	0.60 (0.37–0.96)	0.034	0.045
GBU4-5 (≥45% reduction)	0.50 (0.29–0.84)	0.009	0.059	0.52 (0.30–0.89)	0.017	0.034
CAGE (≥50% reduction)	0.45 (0.27–0.75)	0.003	0.039	0.47 (0.28–0.79)	0.005	0.020
NY-ESO-1 (≥50% reduction)	0.65 (0.40–1.07)	0.090	0.195	—	—	—
SOX2 (≥45% reduction)	0.56 (0.33–0.96)	0.035	0.114	0.59 (0.34–1.04)	0.070	0.070
MUC1 (≥40% reduction)	0.85 (0.51–1.41)	0.540	0.780	—	—	—
APE1 (≥40% reduction)	0.90 (0.55–1.47)	0.680	0.884	—	—	—
PGP9.5 (≥50% reduction)	0.96 (0.58–1.59)	0.860	0.931	—	—	—
GAGE7 (≥50% reduction)	1.00 (0.60–1.67)	1.000	1.000	—	—	—
MAGE1 (≥50% reduction)	1.05 (0.64–1.74)	0.840	0.991	—	—	—
Age (per 1-year increase)	1.05 (1.00–1.09)	0.052	0.135	—	—	—
Stage II vs Stage I	1.40 (0.90–2.35)	0.110	0.204	—	—	—
Sex (female vs male)	0.82 (0.48–1.39)	0.460	0.748	—	—	—
Smoking (current/ex vs never)	1.08 (0.58–2.02)	0.800	0.888			
Hypertension	1.02 (0.54–1.93)	0.960	0.960			
Diabetes	1.27 (0.64–2.53)	0.490	0.700			
Charlson ≥ 2	1.33 (0.70–2.52)	0.380	0.570			

## 5 Discussion

TAA‑associated autoantibodies are recognized as promising biomarkers for cancer detection, progression, and prognosis. In our study, dynamic shifts in autoantibody levels after lung‑cancer surgery provided valuable insights into patient outcomes. Postoperative decreases in specific autoantibodies – p53, GBU4‑5, CAGE, and NY‑ESO‑1 – may serve as potential prognostic markers for lung cancer.

Immediately after thoracic resection, tissue-damage-associated molecular patterns trigger a systemic cytokine surge (IL-6, IL-8) and a brief lymphopenia/NK-cell dip that can last several days; this “danger-signal” phase is now recognized as a normal, self-limiting response that resolves more quickly after minimally invasive surgery [25]. Concomitantly, myeloid-derived suppressor cells and regulatory T cells expand, creating a transient window of immunosuppression during which residual tumor cells may evade surveillance [26]. Over subsequent weeks, adaptive immunity rebounds, cytotoxic T/NK activity is restored and – provided that viable tumor antigen has been removed – circulating autoantibody titers fall. Optimized peri-operative strategies can blunt the early inflammatory spike and accelerate immune recovery, reinforcing our observation that durable reductions in p53, NY-ESO-1, and related autoantibodies are most evident once the pro-inflammatory milieu has subsided and effective tumor immunity is re-established [27].

The autoantibody panel evaluated in this study – P53, MUC1, NY‑ESO‑1, APE1, PGP9.5, SOX2, GBU4‑5, GAGE7, CAGE, and MAGE1 – was purposefully chosen for their well‑documented roles in lung‑cancer biology and host immune surveillance. These TAAs are often over‑expressed in lung tumors, triggering robust immune responses that yield measurable circulating autoantibodies. Anti‑P53 autoantibodies, for instance, mirror mutations in the tumor‑suppressor gene and are reliable markers of tumor activity and recurrence [23,28]. Similarly, the cancer‑testis antigens NY‑ESO‑1 and MAGE1 are strongly expressed in lung cancer, and their corresponding autoantibodies correlate with poor prognosis and elevated recurrence risk [13,21]. Incorporating MUC1 – a glycoprotein whose aberrant glycosylation drives adenocarcinoma progression – broadens the panel’s diagnostic utility [22]. Autoantibodies such as GBU4‑5 and CAGE have also demonstrated predictive value for disease recurrence [19,24]. By integrating markers with proven clinical relevance, this panel allows nuanced assessment of tumor dynamics, immune response, and recurrence risk. Longitudinal monitoring has shown that postoperative declines in autoantibodies such as P53, NY‑ESO‑1, and CAGE are associated with favorable outcomes, underlining their potential in personalized postoperative care [29].

Extensive evidence now shows that TAA autoantibodies predict key clinical outcomes, including recurrence‑free and OS. Their value as lung‑cancer biomarkers was first recognized nearly two decades ago [13]. Subsequent studies have linked autoantibodies to cancer‑testis antigens such as MAGE to both diagnosis and prognosis [21] and have demonstrated that TAA‑directed autoantibodies predict responses to immune‑checkpoint inhibitors in NSCLC [23]. A highly predictive autoantibody panel has likewise been reported to guide postoperative management in early‑stage NSCLC [19]. In line with these findings, our study identifies postoperative declines in P53 and MAGE autoantibody titers as markers of lower recurrence risk and improved survival, underscoring the close relationship between immune surveillance and clinical outcome.

High circulating anti-p53 titers almost invariably originate from conformationally altered (mutant) p53 that accumulates in tumor cells; these mutants not only mark heavy tumor burden but actively dampen innate‐immune sensors such as cGAS-STING and NF-κB signaling pathways [30,31]. Surgical resection removes the antigen source, precipitating a rapid fall in anti-p53 antibodies; the concurrent relief of mutant-p53-mediated immunosuppression helps restore antitumor immunity, a pattern echoed in NSCLC cohorts where persistently high anti-p53 titers predicted early relapse and shorter survival [32].

NY-ESO-1, in contrast, is a highly immunogenic cancer-testis antigen whose expression surges with hypomethylation in proliferating tumor cells. Robust humoral and CD8⁺ T-cell responses arise once the antigen load is high, and antibody levels track closely with viable tumor mass. Multicenter data show that durable post-therapy declines in NY-ESO-1 antibodies signify minimal residual disease and translate into longer disease-free and OS [17,33]. Moreover, simultaneous NY-ESO-1-specific antibody and CD8⁺ responses correlate with superior outcomes in immunotherapy, suggesting that once tumor antigen is removed, falling antibody titers accompany a shift from chronic antigenic stimulation to effective effector-T-cell surveillance [34]. Together, these observations provide a biological rationale for the survival advantage observed in our cohort when p53 or NY-ESO-1 autoantibodies decline.

The robust associations we observed between postoperative declines in specific autoantibodies and improved outcomes support integrating autoantibody profiling into routine surveillance. Persistently elevated titers – particularly of CAGE or GBU4‑5 – could trigger closer monitoring or adjuvant therapy to address residual disease or micrometastases. Because these assays are blood‑based, they offer a non‑invasive, cost‑effective complement to imaging for tracking tumor dynamics after surgery. Our findings therefore underscore the clinical value of incorporating autoantibody testing into standard follow‑up protocols to enhance recurrence detection and improve survival in lung‑cancer patients.

As certain autoantibodies (e.g., SOX2 at the 3-month time point) exhibited changes approaching but not reaching conventional significance (*p* < 0.05). While these borderline results should be interpreted with caution, they may still have clinical relevance, particularly in a pilot or exploratory context. Consequently, while results like the SOX2 trend at 3 months are not definitively significant in this study, they underline the importance of continued investigation into the dynamic behavior of autoantibodies post-surgery. Clinicians may still find value in monitoring these markers as part of a broader risk assessment panel, pending validation in more extensive cohorts.

Although our results are promising, they are derived from a single-center study and have not been validated in an independent patient cohort. This limitation restricts the generalizability of our findings, as patient demographics, tumor biology, and treatment protocols may vary between institutions and geographic regions. To overcome this, future research should incorporate prospective, multi-center trials or external validation sets to confirm the prognostic significance of these autoantibody changes in broader and more heterogeneous populations. One notable limitation of this study is the absence of a non-malignant control group (e.g., individuals with benign lung conditions or healthy volunteers). Such a comparator could have clarified whether the baseline autoantibody levels observed in our lung cancer cohort diverge significantly from those in non-cancer populations. Although the primary objective was to investigate longitudinal changes post-surgery and their prognostic value, a proper control group would enhance our ability to discern disease-specific patterns and improve specificity for lung cancer. Future larger studies or multi-center collaborations should include an appropriate control arm to better contextualize baseline autoantibody titers and refine their clinical interpretation. Moreover, the absence of standardized cut‑off thresholds for autoantibody positivity remains a major obstacle, impeding cross‑study comparisons and limiting reproducibility. Conducting larger, methodologically harmonized studies and adopting consensus diagnostic criteria will strengthen the clinical applicability and translational potential of autoantibody profiling in lung‑cancer management. Expanding analyses to include additional TAAs could uncover biomarkers with complementary diagnostic and prognostic value [29]. Developing a comprehensive, multi‑marker “autoantibody signature” would further enhance risk stratification and enable truly personalized patient care. Pursuing these directions will help establish standardized, clinically actionable tools to improve lung‑cancer monitoring and therapeutic outcomes.

Our study demonstrates that longitudinal assessment of autoantibody levels before and after lung‑cancer surgery has significant predictive value for recurrence, metastasis, and OS. Tracking these changes enables tailored therapeutic strategies based on individual risk profiles. Collectively, the evidence supports integrating autoantibody monitoring into routine postoperative care for lung‑cancer patients to improve outcomes through more precise, personalized management.
